# Practitioners, Consultants, and Fees

**Published:** 1911-12-02

**Authors:** 


### The Hospital

The Workers' Newspaper of Administrative Medicine and Institutional Life, Administration, National Insurance and Health.

No. 1325, Vol. LI. SATURDAY, DECEMBER 2ND, 1911. PRICE ONE PENNY.

### PRACTITIONERS, CONSULTANTS, AND FEES.

From time to time a case is discovered of the practice euphemistically known as "dichotomy," and attention is thereby directed, both among the public and in the medical profession, to the respective emoluments of the general practitioner and the consultant, and to the possibility of corrupt dealings between them. To the commercial mind corruption is an inevitable result of the system whereby payment for consultations is regulated; and no denial, however earnest, will avail to persuade some men engaged in "business" that such corruption is in fact rare or non-existent. Even among the classes who are less frequently brought into contact with the methods of "the City" an uneasy suspicion is not uncommon that some redistribution of fees is taking place behind the patient's back whenever a consultation with a specialist has been held.

The system—for want of a better word—which breeds this distrust in some people is, it must be admitted, a standing temptation to dishonesty. For a consulting surgeon often receives for performing an operation, which may be quite simple or may be extremely difficult, a sum of money equal to that which the general practitioner would ask for daily attendance throughout a year. In other words, the surgeon is twenty times and more overpaid for the actual time he spends (which alone can be appreciated by the patient) compared with the general practitioner. Now the choice of a surgeon in most cases is made on the recommendation of the practitioner, who collects for the former and hands over to him his fee, and is thus in the position of agent in respect of him. Hence it might arise—indeed has occasionally arisen—that the practitioner successfully demands a substantial commission from the surgeon to whom he has introduced the patient. This is the practice that the Americans have named dichotomy; a term which is supposed to mean a splitting, or division.

Does dichotomy—that is to say, the custom of giving or taking secret commissions of this sort—exist in England? The answer can be given confidently and conscientiously. The practice does exist, but only to an extremely limited extent; and the general sense of the profession revolts so wholly against it that any who admitted it would be immediately ostracised by the great bulk of their fellow practitioners and fellow consultants. This may seem incredible to some of those familiar only with the business "morality" of commercial circles; but it is nevertheless perfectly true, and it is a remarkable tribute to the high standard of honour in the profession, that this temptation to illicit commissions is so universally resisted. The only instance which has come under the notice of the present writer was a minor degree of the practice, though the principle was equally indefensible. A well-known surgeon received a fee of one hundred guineas for performing an operation. He paid the anæsthetist three guineas, and his assistant two guineas. The practitioner in charge of the case was present at the operation, and was dressed and washed in readiness to assist if necessary; however, he actually did nothing whatever, and the surgeon paid him five guineas for his services.

As regards countries other than Great Britain, the same does not everywhere hold good. Thus not many weeks ago (The Hospital, September 30, 1911, p. 667) attention was drawn to a play, then being acted in Paris, wherein the division of a very large fee between the operator and the family doctor is the underlying *motif* of a very gruesome tragedy. The play evoked from the "Syndicat Medical de Paris" a resolution expressing the detestation of that body for the whole principle and practice of dichotomy, and a declaration that this is rare in France, but the resolution may have been accepted with faint tinges of doubt and suspicion by the Parisian public. Nevertheless, the high level of professional honour in France is sufficient guarantee that the views of the "Syndicat" are those of the majority of French practitioners, as they are those of the general body of doctors in all countries and in all ages.

Of the United States the same general conclusion, it may be surmised, is not so certain. The *New York Herald* ventilated this question lately, and obtained expressions of opinion from eight or nine leading consultants in that city. Most of them admit (and deplore) that, in the West, dichotomy is rampant, but say it is not common in New York. The President of the Academy of Medicine admits that the evil exists in New York, but adds that any man guilty of it is expelled from the organisation. One of the authorities consulted even appears to condone dichotomy. The men out West may resent the imputations of their Eastern colleagues, and may retort with counter charges; but at least enough has transpired to show that dichotomy is not so very rare in America as it is in some other countries. The practical lesson is one which the public will do well to lay to heart—namely, that the labourer is worthy of his hire; and that a properly remunerated general practitioner runs little risk of falling into temptation of this kind, whereas one whose accounts are paid grudgingly or not at all may possibly do so. Medical men are only human, and human nature is notoriously weak. The astonishing thing is, not that occasional instances of illicit dichotomy should occur, but that this detestable practice is no more common than it is here in good faith represented to be.

